# Application of Ferric–Graphene Quantum Dot Complex for Evaluation and Imaging of Antioxidants in Foods Based on Fluorescence Turn-Off–On Strategy

**DOI:** 10.3390/antiox14091034

**Published:** 2025-08-22

**Authors:** Mahmoud El-Maghrabey, Aya Yamamichi, Ali Abdel-Hakim, Naoya Kishikawa, Naotaka Kuroda

**Affiliations:** 1 Department of Analytical Chemistry for Pharmaceuticals, Graduate School of Biomedical Sciences, Nagasaki University, 1-14 Bunkyo-machi, Nagasaki 852-8521, Japan; dr_m_hamed@mans.edu.eg (M.E.-M.); ali.hassan@fop.usc.edu.eg (A.A.-H.); 2 Department of Pharmaceutical Analytical Chemistry, Faculty of Pharmacy, Mansoura University, Mansoura 35516, Egypt; 3 Department of Analytical Chemistry, Faculty of Pharmacy, University of Sadat City, Monufia 32897, Egypt

**Keywords:** fluorescence imaging, antioxidant assay, antioxidant distribution profile, graphene quantum dots, Fe^3+^-reducing ability

## Abstract

Antioxidants have drawn much interest owing to their capacity to shield the human body from reactive oxygen species (ROS). Therefore, it is essential to develop a quick and easy assay for the evaluation of antioxidants and for imaging their distribution in food. Herein, we describe a fluorescence measurement platform for assessing and visualizing antioxidant capacity. Our method is based on using the composite of graphene quantum dots (GQDs) with Fe^3+^ (Fe^3+^-GQDs) as a reagent for evaluating and imaging the antioxidant capacity in foods using a fluorescence turn-off–on strategy. The fluorescence of GQDs was found to be selectively quenched with Fe^3+^ at pH 3.5. Upon addition of an antioxidant, Fe^3+^ is reduced to Fe^2+^, and the fluorescence of GQDs is regained. Next, we investigated the fluorescence intensity after the reaction of Fe^3+^-GQDs with seven typical antioxidants, and it showed excellent sensitivity down to 0.60 µM of antioxidant. Next, using Fe^3+^-GQDs as a reagent, we developed a paper-based fluorescence imaging method for antioxidants in foods. Furthermore, we analyzed the distribution of antioxidant capacity on cucumber and carrot slices (tips, central parts, and shoulders). Next, the antioxidant capacity of cucumber and carrot slice extracts was measured, and the results were consistent with the fluorescence imaging results of the intact slices.

## 1. Introduction

Antioxidants such as l-ascorbic acid protect the body from oxidative stress caused by reactive oxygen species [1]. It is known that oxidative damage to biological components causes various diseases such as cancer [2], liver disease [3], Alzheimer’s disease [4], aging [5], arthritis [6], inflammation [7], diabetes [8], Parkinson’s disease [9,10], atherosclerosis [11], and acquired immune deficiency syndrome [12]. Therefore, antioxidants are valuable compounds for living organisms to maintain biological activities [13]. In recent years, many functional foods and nutritional foods claiming antioxidant effects have been put on the market, and their health maintenance effects have actually been recognized on the basis of chemical evidence. Therefore, there is a need to develop an efficient method for the quantification of antioxidants and an analytical method for antioxidant capacity that can be applied to the search for valuable antioxidants in foods. In addition, imaging methods that can visualize the distribution of antioxidants in foods are useful for finding effective antioxidants in foods. However, most of the currently used antioxidant capacity measurement methods are based on the homogenate of crushed food samples, which causes the loss of positional information on the location of the superior antioxidants in the samples.

So far, the 2,2-diphenyl-1-picrylhydrazyl (DPPH) method [14,15], the 2,2′-azino-bis(3-ethylbenzothiazoline-6-sulfonic acid) (ABTS+) method [16], the ferric reduction antioxidant power (FRAP) method [15,17,18], and the oxygen radical absorbance capacity (ORAC) method [15,19] have been used to evaluate antioxidants in natural products. However, the DPPH and ABTS+ methods are somewhat unreliable because they measure the ability to remove free radicals and cannot distinguish the conversion of free radicals to other substances that are not related to these reactions. The FRAP method, which measures the coloration caused by the reduction of Fe^3+^ to Fe^2+^, has the disadvantage of poor sensitivity and selectivity as it is based on absorption spectrophotometry. The ORAC method is a method to measure the delay of the fluorophore decomposition reaction caused by reactive oxygen species brought by antioxidants, but it has the disadvantage that the reaction takes several minutes to several hours to complete.

**Scheme 1:**
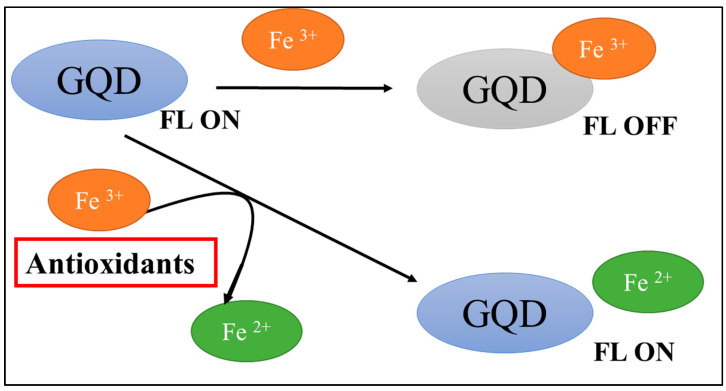
Mechanism of the antioxidant capacity fluorescence assay based on Fe^3+^-GQDs.

Our research group is focusing on quinone’s versatile applications in analytical chemistry [20,21,22,23,24]. Recently, we have developed the first method for tissue distribution imaging of antioxidants in food samples using quinone as a redox and chemiluminescence probe [25]. However, this method could not detect some antioxidants, such as Trolox, and its applicability is not widespread due to the unavailability of chemiluminescence measuring devices in many laboratories. Fluorometry is one of the most widely used sensitive techniques in analytical chemistry and imaging [26,27,28]; however, it necessitates a derivatization reaction to analyze non-fluorescent molecules [29]. Hence, in this study, we developed a derivatization-free fluorescence quantification method for antioxidants using the properties of graphene quantum dots (GQDs) and a fluorescence imaging method for visualizing and analyzing the distribution of antioxidants in foods. GQDs are carbon-based nanoparticles with diameters in the nanometer range and have attracted much interest since they were first reported by Xiaoming Sun et al. in 2008 [30]. GQDs disperse well in water and have excellent properties, such as low toxicity compared to inorganic quantum dots [27,28,31,32]. In addition, GQDs show size-dependent fluorescence, do not fade, and their fluorescence is known to be stable [33]. Recently, bioimaging and fluorescence quantification methods [31,34,35] using GQDs have been developed based on these properties. Although GQDs are themselves fluorescent, their fluorescence is known to be quenched by the addition of other substances, such as Fe^3+^ [31,36]. In this case, the presence of antioxidants reduces Fe^3+^ to Fe^2+^, and as a result, the quenching of GQDs is suppressed and the fluorescence intensity increases (Scheme 1). In this study, we focused on the determination of water-soluble antioxidants. Although both classes of antioxidants (water-soluble and fat-soluble) are able to reduce Fe^3+^ to Fe^2+^ [37], the fat-soluble antioxidants need additional extraction steps. Therefore, the fat-soluble antioxidants would not reduce Fe^3+^ to Fe^2+^ upon direct analysis and imaging of real samples. We considered whether the increase in fluorescence intensity due to antioxidants could be evaluated as the strength of antioxidant capacity. Since this method is a fluorescence analysis method, it is expected to have higher sensitivity and selectivity than the absorption spectrophotometry-based method. In addition, the reaction of antioxidants with the Fe^3+^-GQD complex does not require long incubation time, and the analysis of antioxidants can be performed relatively quickly. Furthermore, the fluorescence of GQDs is stable, and the maximum wavelength of the fluorescence is relatively long (505 nm), which facilitates its application to fluorescence imaging.

At first, a fluorescence quantification method for antioxidants using Fe^3+^-GQD complexes was developed. Typical antioxidants such as l-ascorbic acid were selected as samples, and the analytical performance in terms of sensitivity, quantitation, and reproducibility was evaluated after studying the optimum reaction conditions. Next, the method was extended to assess the antioxidant capacity and imaging using a fluorescence imaging system, Chemidoc. Finally, the developed imaging method was applied to evaluate the antioxidant capacity of food samples and visualize the distribution of antioxidants on vegetable slices.

## 2. Materials and Methods

### 2.1. Chemicals, Reagents, and Solutions

Graphene quantum dots (GQDs), aqua green luminescent, 1 mg/mL in H_2_O, and (+)-catechin were purchased from Sigma-Aldrich (St. Louis, MI, USA). Ferric nitrate, acetic acid, l-ascorbic acid, Trolox, tris(hydroxymethyl)aminomethane (Tris), lithium nitrate, barium chloride, zinc acetate, cobalt chloride, nickel chloride hexahydrate, and strontium chloride were from Wako Pure Chemical Ind., Ltd. (Osaka, Japan). Sodium acetate, potassium chloride, sodium chloride, hydrochloric acid, calcium chloride, gallic acid, caffeic acid, pyrogallol, and dithiothreitol (DTT) were purchased from Nacalai Tesque Co., Ltd. (Kyoto, Japan). Magnesium chloride anhydrous, copper (II) acetate 1-hydrate, and manganese chloride 4-hydrate were from Kishida Chemical Co., Ltd. (Osaka, Japan). Iron (II) chloride, anhydrous, was from the Kojundo Chemical laboratory (Saitama, Japan).

Tris-HCl buffer (30.0 mM, pH 7.0, adjusted using HCl) and acetate buffer (30.0 mM, pH 3.5, adjusted using acetic acid) were used.

### 2.2. Instrumentation and Materials

The fluorescence study was performed with an RF-1500 Shimadzu fluorometer (Kyoto, Japan). The sample cell was a 1.0 cm quartz cell, and the slit widths of the excitation and emission monochromators were set at 10 nm. The fluorescence imaging of the food samples and paper-based assays was performed using the ChemiDoc^TM^ Touch Imaging System (Bio-Rad Laboratories, Hercules, CA, USA) using super-sensitivity mode. The illumination mode used was standard trans-UV, 302 nm. The used emission filter was 590 nm/110 nm. Fourier transform infrared (FT-IR) spectrum measurements were performed using an IR Affinity-1 FT-IR spectrophotometer from Shimadzu (Kyoto, Japan) in the wavenumber range of 4000–750 cm^−1^. The fluorescence lifetime was measured using a Quantaurus-Tau^®^ Fluorescence lifetime spectrometer (C16361 series, Hamamatsu Photonics, Shizuoka, Japan) over a time range of 0.0–50.0 ns, using a 405 nm LED light as the excitation source. The device determines the average lifetime using exponential functional fitting and spectrum analysis. In the fabrication of the paper-based antioxidant assay, the filter paper used was No. 6 (ADVANTEC, Tokyo, Japan). The pH was measured and adjusted on the F-71 Horiba pH meter (Kyoto, Japan). For sonication and mixing of the solutions during reagent solution preparation and sample extraction, a US-102N Sango ultrasonic cleaner and V-1 plus vortex mixer (Funakoshi Co., Ltd., Tokyo, Japan) were used, respectively.

### 2.3. Spectrofluorometric Measurement of Antioxidants Using Fe^3+^-GQD Composite

All the fluorescence measurements were conducted at room temperature. To 0.6 mL of GQDs (30 μg/mL), 0.6 mL of acetate buffer (30 mM, pH 3.5), and 0.3 mL of the aqueous antioxidant solution were added, followed by the addition of 0.3 mL of aqueous Fe(NO_3_)_2_. Next, the green fluorescence of the GQDs was measured at 505 nm after excitation at 430 nm. The fluorescence intensities were plotted vs. the antioxidant concentrations to construct the calibration curves for the studied antioxidants.

The same procedure was followed to determine the antioxidant content in juice samples (lemon and apple juice), but the sample (0.3 mL, after diluting the juice samples 20 times) was added instead of the aqueous antioxidant solution. Then, the total antioxidant capacity was determined from the constructed calibration curve.

### 2.4. Procedure for the Paper-Based Fluorescence Image Assay of l-Ascorbic Acid

Filter papers with diameters of 6 mm were prepared by punching the filter paper with a 6 mm puncher. To the prepared filter paper, 2.5 μL of GQDs (1 mg/mL) was added and left at room temperature for 10 min. Next, 2.5 μL of 5.0 mM Fe(NO_3_)_2_ in acetate buffer (30 mM, pH 3.5) was added to the filter paper and left at room temperature for 10 min. Afterward, 2.5 μL of l-ascorbic acid (0.05–2.0 mM) was added. Next, the green fluorescence of the GQDs on the filter paper was measured at (480–600 nm) after excitation at 302 nm using the ChemiDoc^TM^ imaging system. Next, the gray value of the fluorescence from each filter paper with different l-ascorbic acid concentrations was obtained using ImageJ software, version 1.52a. The gray values were plotted vs. the l-ascorbic acid concentration to construct the calibration curve.

The vitamin C drink C1000 Vitamin Lemon, a product of House Wellness Foods (Hyogo, Japan), was used. The concentration of l-ascorbic acid in the C1000 Vitamin Lemon supplement was measured using paper-based fluorescence imaging through dilution of the supplement till reaching concentrations of 0.1 mM, 1.0 mM, and 2.0 mM l-ascorbic acid; then, 2.5 μL of these solutions were applied to the filter paper impregnated with GQDs and Fe(NO_3_)_3_. Next, the l-ascorbic acid recovery and nominal content in the supplement were calculated using the regression equation of the calibration graph of the paper-based assay of l-ascorbic acid.

### 2.5. Antioxidant Distribution Imaging in Vegetable Sections

Sections of cucumber with a thickness of 3 mm were cut from the tip and the central part of the cucumber. Slices of carrot with a thickness of 3 mm were also cut from the tip and shoulder of the carrot. Next, the slices were impregnated with 0.2 mL of GQDs (1 mg/mL) and left at room temperature for 10 min. Then, they were impregnated with 0.2 mL 5.0 mM Fe(NO_3_)_2_ in acetate buffer (30 mM, pH 3.5) and left at room temperature for 10 min. Afterward, the fluorescence of the GQDs on the vegetable slices was measured at 480–600 nm after excitation at 302 nm using the ChemiDoc^TM^ imaging system.

Moreover, the total antioxidant capacities of the central and tip part of the cucumber and the shoulder and tip part of the carrot were measured using the paper-based fluorescent assay. These parts were crushed by a mixer. Next, 0.2 g of the crushed samples were transferred into Eppendorf tubes and then extracted with 1.0 mL of water using sonication for 5 min. Afterward, the Eppendorf was centrifuged at 2200× *g* for 5 min. Then, the upper layer was taken and measured using the procedure described in Section 2.4.

## 3. Results

GQDs are themselves fluorescent, but their fluorescence is quenched by the addition of metal ions such as Fe^3+^. It was found that the fluorescence of GQDs was quenched selectively with Fe^3+^ at pH 3.5. In the presence of antioxidants, Fe^3+^ is reduced to Fe^2+^, which suppresses the quenching of GQDs, so that they regain their fluorescence. Based on these results, we attempted to develop a fluorescence quantification method for antioxidants using a Fe^3+^-GQD complex as a reagent.

### 3.1. Characterization of the Used GQDs

**Figure 1:**
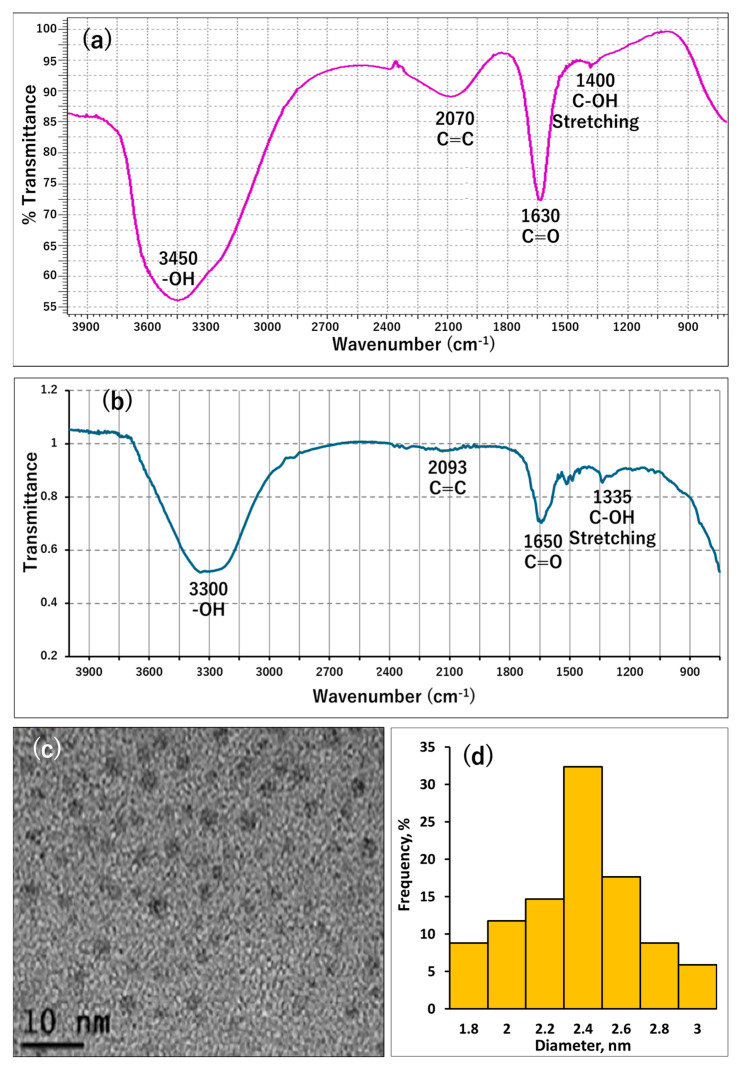
Characterization of GQDs; where (**a**) shows the FT-IR spectrum of GQDs, (**b**) shows the FT-IR spectrum of GQDs in the presence of Fe^3+^, (**c**) shows a TEM image of GQDs, and (**d**) shows a histogram of the GQDs’ particle diameter distribution.

Various microscopic and spectroscopic methods were used to analyze the surface structure and spectral characteristics of the GQDs. The surface groups of the GQDs were inspected using FT-IR spectrum measurement. The FT-IR spectrum, illustrated in Figure 1a, shows that the GQDs have on their surface the following functional groups: OH (stretching peak at 1400 cm^−1^ and broad peak at 3450 cm^−1^), C=O (sharp peak at 1630 cm^−1^), and aromatic C=C (peaking at 2070 cm^−1^) [38]. This could be interpreted as the GQDs consisting of a polyaromatic core with phenolic, carbonyl, and carboxylic groups at their surface, enhancing their hydrophilic properties and explaining their water solubility and stability. Additionally, these groups could interact with metal ions, affecting the GQDs’ fluorescence properties. Then, the FT-IR spectrum of GQDs in the presence of Fe^3+^ was measured (Figure 1b). It was found that the binding of Fe^3+^ to GQDs caused notable changes in the FTIR spectrum, where the OH peak (stretching peak) decreased from 1440 cm^−1^ to 1335 cm^−1^ and the broad peak at 3450 cm^−1^ decreased its broadness and intensity and shifted to 3300 cm^−1^. Additionally, the C=O peak (sharp peak) became much less sharp and shifted to 1650 cm^−1^. Moreover, the aromatic C=C peak (peaking at 2070 cm^−1^) was largely decreased and quenched. These results indicate the binding of Fe^3+^ to the surface OH, C=O of the carboxylic group, and the electronic interaction between aromatic C=C and Fe^3+^, which is similar to what has been previously reported in studies [39,40,41]. Next, the GQDs’ size was inspected using transmission electron microscopy (TEM), and the shape and size of the GQDs are shown in Figure 1c,d. The GQDs possess a spherical shape with a narrow distribution range of 1.8–3.0 nm and a mean size of 2.4 nm.

### 3.2. Fluorescence Monitoring of Fe^3+^ Interaction with GQDs

**Figure 2:**
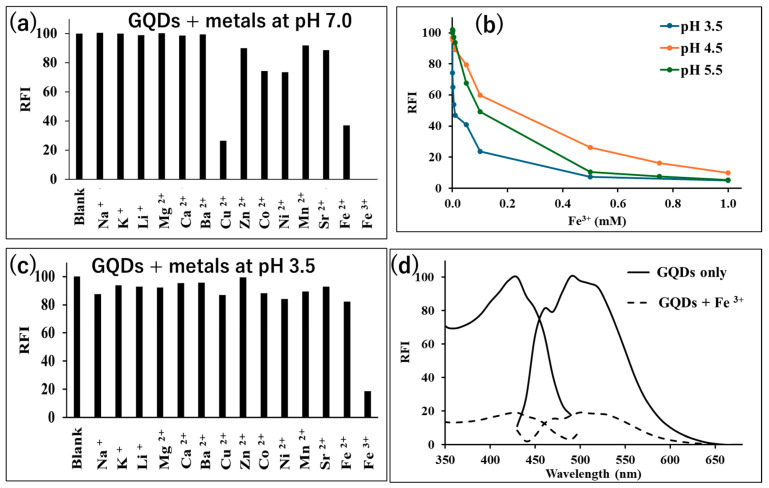
Metals’ interactions with GQDs and selectivity study; where (**a**) shows the RFI intensity of GQDs (10 μg/mL) alone (blank) and after their interaction with different metal ions (2.0 mM) at pH 7.0 (Tris-HCl buffer) [*n* = 3]; (**b**) effect of pH of acetate buffer on RFI of GQDs (10 µg/mL) after addition of Fe^3+^ (0–1.0 mM), where λex = 430 nm and λem = 505 nm [*n* = 3]; (**c**) RFI of GQDs (10 μg/mL) in sodium acetate buffer (30 mM, pH 3.5) in the presence and absence of different metal ions (2.0 mM) [*n* = 3]; and (**d**) the fluorescence spectra of GQDs (10 μg/mL) in acetate buffer (30 mM, pH 3.5) in the presence and absence of Fe^3+^ (2.0 mM).

At first, we investigated the change in fluorescence intensity of GQDs due to the addition of metal ions under neutral conditions using Tris-HCl buffer (pH 7.0, 30 mM). The results are illustrated in Figure 2a, where the most significant fluorescence quenching was observed when Fe^3+^ aqueous solution was added. In addition, fluorescence quenching was observed when Cu^2+^ and Fe^2+^ aqueous solutions were added. In order to increase the selectivity of GQDs towards Fe^3+^, the interaction of Fe^3+^ with GQDs under low-pH conditions was tested, as the bond between hydroxyl groups on the GQDs’ surface and Fe^3+^ is stronger than the bond with other metal ions such as Cu^2+^, Co^2+^, and Ni^2+^ [42]. By adding an aqueous Fe^3+^ solution to an aqueous GQD dispersion containing a sodium acetate buffer and measuring the fluorescence intensity, we investigated the pH at which GQDs and Fe^3+^ interact most strongly (Figure 2b). As a result, we confirmed that the fluorescence of GQDs was most markedly quenched as the Fe^3+^ concentration increased under the condition of pH 3.5. Therefore, sodium acetate buffer solution at pH 3.5 was used in the subsequent experiments.

Next, the selectivity study was repeated under pH 3.5, and as shown in Figure 2c, the GQDs showed exceptional selective fluorescence quenching upon interaction with Fe^3+^ without any interference from other metals. The fluorescence spectra of the GQDs alone and upon their interaction with Fe^3+^ at pH 3.5 are shown in Figure 2d.

**Figure 3:**
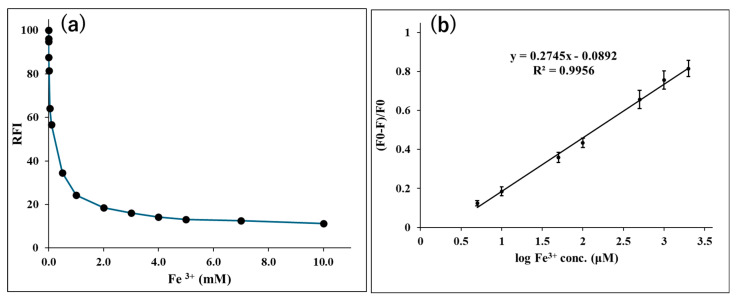
Fluorescence monitoring (λex/λem = 430/505 nm) of the interaction between Fe^3+^ and GQDs; where (**a**) shows the effect of the concentration of Fe^3+^ on RFI of GQDs (10 µg/mL) in sodium acetate buffer (30 mM, pH 3.5) after addition of Fe^3+^ (0–10.0 mM) [*n* = 3]; and (**b**) shows the calibration curve of the log concentration of Fe^3+^ vs. relative quenching (F0 − F)/F0) [*n* = 3].

Afterward, the quenching of GQDs upon their interaction with Fe^3+^ at a wide concentration range of 0 to 10.0 mM was investigated, and the results are shown in Figure 3a. As the concentration of Fe^3+^ increases, the fluorescence of GQDs is increasingly quenched till reaching 2.0 mM Fe^3+^. Increasing the Fe^3+^ concentration to more than 2.0 mM did not cause more significant fluorescence quenching. Consequently, a calibration curve was constructed for Fe^3+^ through plotting Log (Fe^3+^ μM) vs. the relative quenching intensity ((F0 − F)/F0), where F0 and F are the GQDs’ fluorescence intensities in the absence and presence of Fe^3+^. The method showed good linearity (r = 0.998) over the range of 5.0 to 2000 μM Fe^3+^.

### 3.3. Study of the Mechanism of Fluorescence Quenching of GQDs After Interaction with Fe^3+^

**Figure 4:**
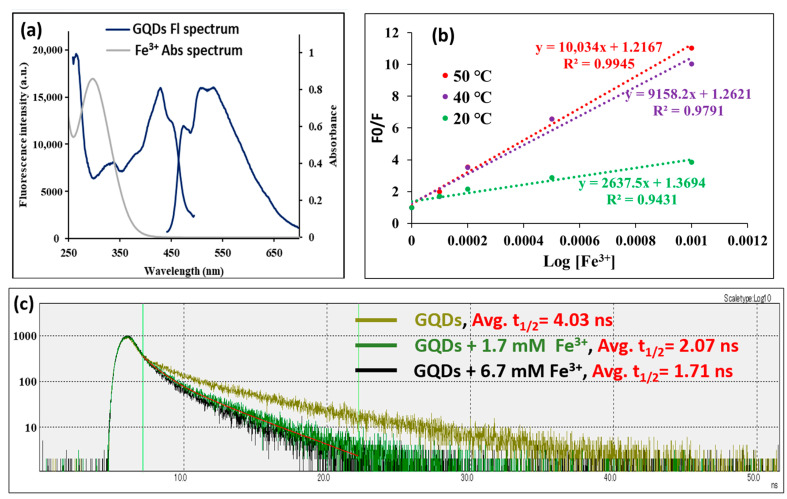
The quenching mechanism; where (**a**) shows the absorbance spectrum of Fe^3+^ and the fluorescence spectrum of GQDs, (**b**) the Stern–Volmer plot for Fe^3+^ interaction with GQDs at three different temperatures [*n* = 3], and (**c**) the fluorescence lifetime curve for GQDs, either alone or in the presence of Fe^3+^ (1.7 mM and 6.7 mM), from 0.0 to 50.0 ns. The red line shows the exponential function fitting of GQDs + 1.7 mM Fe^3+^ as an example for the fitting method carried out for every spectrum.

The mechanism of fluorescence quenching of GQDs by Fe^3+^ could be the inner filter effect, static quenching due to ground state complexation, dynamic collisional quenching, or a combination of these mechanisms. At first, we investigated whether there is an overlap between the absorption spectrum of Fe^3+^ and the fluorescence spectrum of GQDs. As shown in Figure 4a, it was found that there is no overlap between the two spectra, which excludes the possibility of inner filter-driven quenching. Next, to determine whether the quenching of GQDs with Fe^3+^ is static or dynamic quenching, the effect of temperature on the quenching of the GQDs’ fluorescence was investigated. Raising the temperature amplifies dynamic quenching and reduces static quenching. An investigation using the Stern–Volmer equation was performed to elucidate the primary quenching mechanism by studying the quenching data at different temperatures (20, 40, and 50 °C) and applying the following equation [28]:

F0/F = 1 + K [Q]

K and Q are the quenching constant and quencher concentration, respectively. Upon plotting F0/F against Q, the slope gives the quenching constant (K). The Stern–Volmer plots at three different temperatures are shown in Figure 4b. As the temperature increases, the slope (quenching constant) increases, which demonstrates that the quenching mechanism is dynamic quenching. In dynamic quenching, the fluorescence lifetime should be changed upon the interaction of the quencher with the fluorophore. In order to further prove the dynamic quenching mechanism, the fluorescence lifetime of GQDs alone and in the presence of Fe^3+^ was studied, and the results are shown in Figure 4c. The average fluorescence half-life time (t_1/2_) of GQDs was found to be 4.03 ns; however, upon interaction with increasing concentrations of Fe^3+^ (1.7 and 6.7 mM), the t_1/2_ of GQDs was found to be decreased to 2.07 and 1.71 ns, respectively, in a quencher concentration-dependent manner. As can be seen in Figure 4c, the fluorescence lifetime measurements can be analyzed with a two-component model as the fluorescence decay is non-mono-exponential, meaning it does not follow a simple exponential decay. This often happens when a fluorophore exists in multiple environments or has multiple excited-state decay pathways. Consequently, a first and second t_1/2_ were calculated: the first t_1/2_ was GQDs = 1.02 ns, GQDs + 1.7 mM Fe^3+^ = 0.94 ns, and GQDs + 6.7 mM Fe^3+^ = 0.87 ns; and the second t_1/2_ was GQDs = 5.66 ns, GQDs + 1.7 mM Fe^3+^ = 3.54 ns, and GQDs + 6.7 mM Fe^3+^ = 3.19 ns. From these data, Fe^3+^ also decreases the first and second t_1/2_ in a concentration-dependent manner, with a much greater effect on the second t_1/2_. This means that the long component is more influenced than the short one by Fe^3+^. All these results demonstrate that the quenching of GQDs with Fe^3+^ mainly relies on dynamic quenching, which is similar to previous reports [43].

### 3.4. Measurement of the Antioxidants Using the Spectrofluorometry Method

As shown previously in Figure 2c, the fluorescence of GQDs is selectively and strongly quenched upon interaction with Fe^3+^ at pH 3.5, whereas no other metals, even those closely related to Fe^3+^, including Fe^2+^, quenched the fluorescence of GQDs. As Fe^3+^ is reduced by antioxidants’ reducing properties into Fe^2+^, it is expected that the fluorescence of GQDs will be regained upon the addition of antioxidants to the Fe^3+^-GQD composite.

**Figure 5:**
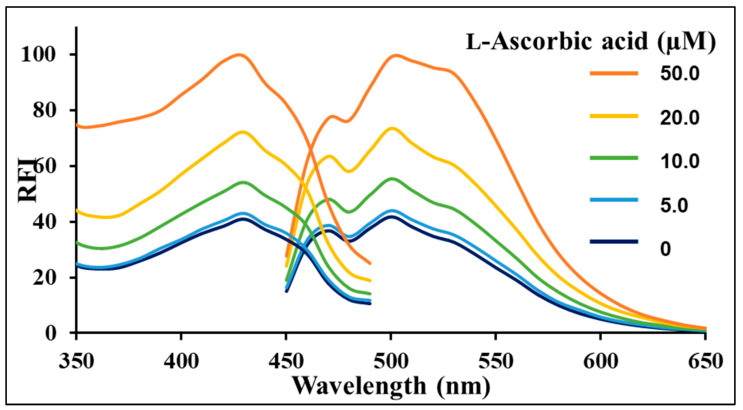
The fluorescence spectra of GQDs (10 µg/mL) with Fe^3+^ (0.3 mM) in sodium acetate buffer (30 mM, pH 3.5) after the addition of different concentrations of l-ascorbic acid (0–50.0 µM) in H_2_O; λex = 430 nm.

Figure 5 shows the changes in fluorescence spectra of Fe^3+^-GQDs when l-ascorbic acid is added as an antioxidant. As shown in the figure, the fluorescence intensity around 505 nm increased with an increasing concentration of l-ascorbic acid. This result suggests that the fluorescence of GQDs is no longer being quenched by the reduction of Fe^3+^ to Fe^2+^ due to the antioxidant effect of l-ascorbic acid.

Based on the previous results, we aimed to develop a fluorescence quantification method for antioxidants based on the regaining of the fluorescence of Fe^3+^-quenched GQDs. For this purpose, the concentration of Fe^3+^ added to the reaction solution was investigated in the range of 0.06 to 0.6 mM. The fluorescence intensity increased quantitatively with increasing l-ascorbic acid concentration for all Fe^3+^ concentrations. Therefore, a calibration curve of l-ascorbic acid was constructed for each Fe^3+^ concentration, and the detection limit was calculated. As a result, the range of quantification became broader as the concentration of Fe^3+^ increased, while the detection limit became larger (Table 1). For a compromise between the detection limit and range, 0.3 mM Fe^3+^ was used in the subsequent studies, as it gave good results in terms of both a wide range of quantification and sensitivity.

The fluorescence intensities of seven typical antioxidant standard solutions were measured according to the procedure in Section 2.3, and calibration curves were generated (Table 2). The results showed that l-ascorbic acid, DTT, and Trolox ranging from 5.0 to 50.0 µM, gallic acid from 2.0 to 20.0 µM, pyrogallol from 5.0 to 20.0 µM, (+)-catechin from 10.0 to 50.0 µM, and caffeic acid in the concentration range of 20.0–100 µM yielded a good linear response between antioxidant concentration and fluorescence intensity, with a coefficient of determination (r^2^) of at least 0.991. Therefore, it was confirmed that this fluorescence quantification method is capable of quantifying a wide variety of antioxidants regardless of their structural differences. The detection limit (3SD/slope) of this method was 0.603–8.23 µM.

The accuracy and precision of the proposed method were investigated for the studied antioxidants. Standard solutions of antioxidants at concentrations corresponding to low, medium, and high concentrations within the calibration range were used. The results are summarized in Table 3. The accuracy of the method, calculated as found%, was acceptable, ranging from 86.08 to 115.8%. The results showed that the reproducibility of the method was less than 8.3% within a day (*n* = 5) and less than 4.2% between days (*n* = 5) (Table 3).

The method was tested to determine the total antioxidant content of samples containing more than one antioxidant. For this, two commercial samples (lemon juice and apple juice) were analyzed using the proposed method. The total antioxidant contents of the samples were determined as shown in Table 4. The same samples were analyzed using the DPPH assay reported in [44] to prove the reliability of the assay. The results of both methods were comparable, proving the applicability and the reliability of the proposed assay.

As our method relies on the ferric-reducing ability of antioxidants, its performance was compared with that of the FRAB assay [45]. The results are shown in Table 5. The sensitivity of the developed method was higher than that of the FRAP method for most of the tested antioxidants, demonstrating the high sensitivity of the proposed fluorometric assay. Additionally, the proposed method showed higher sensitivity to our previously developed chemiluminescence antioxidant assay [25] for gallic acid, and showed lower sensitivity for some antioxidants such as l-ascorbic acid, pyrogallol, and DTT, as shown in Table 5; however, the chemiluminescence method could not analyze the synthetic antioxidant Trolox, demonstrating the broader application of the developed fluorometric method.

### 3.5. Paper-Based Assay of l-Ascorbic Acid Standard

**Figure 6:**
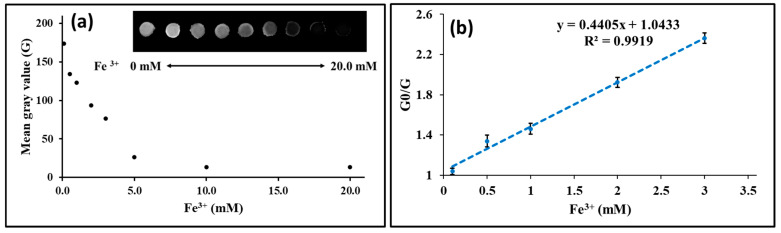
Paper-based fluorescent imaging of Fe^3+^; where (**a**) shows the effect of the concentration of Fe^3+^ on the mean gray value of GQDs, and (**b**) shows the calibration curve for Fe^3+^; and the inset in (**a**) shows an image of filter paper impregnated with GQDs upon addition of increasing concentrations of Fe^3+^ [*n* = 3].

After demonstrating that the fluorescent quantification of antioxidants is possible by using GQDs and Fe^3+^ as reagents, we aimed to extend our method to a paper-based assay. At first, a punched filter paper disc was impregnated with GQDs, and then the imaging of the change in fluorescence intensity of GQDs caused by the addition of Fe^3+^ to the filter paper was measured. After the reaction between GQDs and Fe^3+^ was allowed to react for 10 min, followed by irradiation with excitation light at a wavelength of 302 nm, the fluorescence emitted from a spot of the reaction solution was photographed through a filter (StarBright B520, Bio-Rad Laboratories, Hercules, CA, USA) transmitting light at a wavelength of 590/110 nm (Figure 6a). The images were processed using ImageJ, and the changes in mean gray value (mean intensity value of the selected area in the image) with increasing Fe^3+^ concentration were analyzed and considered as indicators for changes in fluorescence intensity (Figure 6a), where with an increase in the concentration of Fe^3+^, the gray value was found to decrease. The calibration curve was constructed for Fe^3+^ by plotting Fe^3+^ (mM) vs. the value G0/G (Figure 6b). G0 and G are the GQDs’ gray values obtained by ImageJ in the absence and presence of Fe^3+^. The method showed good linearity (r = 0.992) over the range 0.1 to 3.0 mM Fe^3+^. As shown in Figure 6, the fluorescence quenching of GQDs with increasing Fe^3+^ concentration can be effectively visualized.

**Figure 7:**
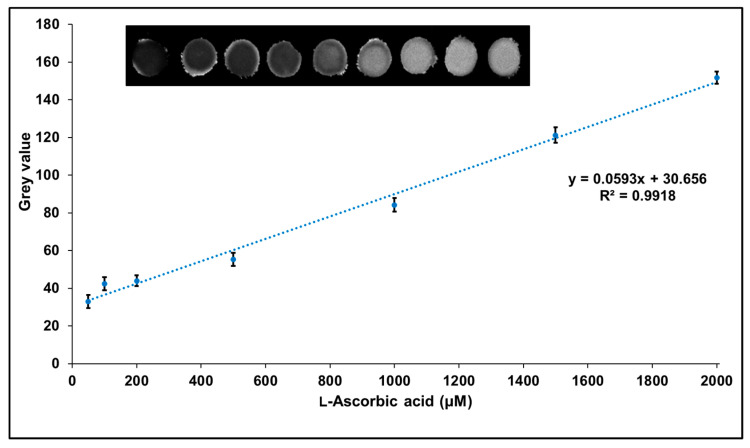
The calibration curve for l-ascorbic acid using the paper-based fluorescent imaging assay, where the inset shows an image of filter paper impregnated with GQDs and Fe^3+^ upon the addition of increasing concentrations of l-ascorbic acid.

Next, we checked whether l-ascorbic acid concentration could be quantified as a fluorescent image by suppressing the quenching of GQD fluorescence by Fe^3+^. As shown in Figure 7, the fluorescence intensity of the spots increases as the concentration of l-ascorbic acid increases. By analyzing this image, a calibration curve for the l-ascorbic acid standard solution was constructed. As shown in Figure 7, an excellent linear relationship with a correlation coefficient of r = 0.996 was obtained between l-ascorbic acid concentration and gray value in the concentration range of 50.0–2000 µM, and the lower detection limit (3SD/slope) was 35.10 µM. Although the sensitivity of the fluorescence imaging method for l-ascorbic acid was lower than that of the fluorometer method mentioned above, it has the advantage that a large number of samples can be measured simultaneously by image capture using very small amounts of reagents and samples.

The accuracy and precision of the proposed paper-based imaging method in determining l-ascorbic acid concentrations were investigated. Standard solutions of l-ascorbic acid at concentrations corresponding to low, medium, and high concentrations within the calibration range were used. The results are summarized in Table 6. The accuracy of the method, calculated as found%, was acceptable, ranging from 93.63 to 115.6%. The results showed that the reproducibility of the method was less than 8.2% within a day (*n* = 5) and less than 7.9% between days (*n* = 5) (Table 6).

### 3.6. Paper-Based Assay of l-Ascorbic Acid in Vitamin C Drink

Next, the fluorescence imaging method we developed was applied to the determination of l-ascorbic acid in vitamin C beverages. The vitamin C drink diluted with water was dropped onto the Fe^3+^-GQDs impregnated on the filter paper to obtain l-ascorbic acid concentrations of 500, 1000, and 1500 µM based on the indicated values in the beverage. The spots were then placed in a fluorescence imaging system, and the fluorescence generated from the spots was photographed and analyzed by ImageJ. The measured l-ascorbic acid content in the vitamin C drink obtained by the fluorescence imaging method was 38.45 ± 2.36 mM (Table 7), which is in good agreement with the indicated value of 40.56 mM. The recovery rate of l-ascorbic acid in this method was more than 92.1%, and the reproducibility was good, with an RSD of less than 6.0%. From these results, it is considered that this imaging method can accurately and precisely quantify l-ascorbic acid without being affected by components other than antioxidants in the beverage sample, such as sugar, honey, acidifier, carbonic acid, coloring, and flavoring agents.

### 3.7. Fluorescence Imaging of Antioxidants in Food Sections and Their Aqueous Extracts

One of the advantages of imaging measurement is its ability to visualize spatial information on the distribution of specific components in a sample. Therefore, we attempted to apply the fluorescence imaging method developed in this study to the visualization and analysis of the distribution of antioxidants in foods.

Sections of cucumbers (central part and tip) and carrots (shoulder and tip) were prepared with a thickness of 3 mm. Next, 0.2 mL of GQDs and 0.2 mL of Fe^3+^ in acetate buffer (30 mM, pH 3.5) were added to these sections and left to stand for 10 min at room temperature. Afterward, the sample sections were placed in a fluorescence imaging system, ChemiDoc, and the fluorescence was measured, as previously mentioned in the experimental section.

**Figure 8:**
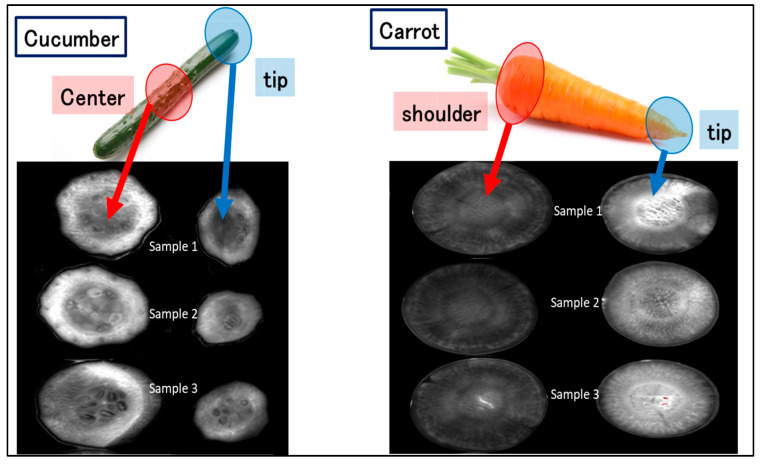
Fluorescence imaging of several cucumber samples (central parts and tip sections) and carrot samples (shoulder and tip sections) after dropping GQDs (1 mg/mL) and Fe^3+^ (5.0 mM) on to them.

Figure 8 shows fluorescence images taken after GQDs and Fe^3+^ (at pH 3.5) were dropped on a central section and a tip section of a cucumber. It was found that strong fluorescence was observed from both the center of the cucumber and at the tip of the cucumber, suggesting that the antioxidants were distributed equally in both parts; however, it was noted that antioxidants are concentrated in the area close to the cucumber skin. In addition, no significant difference was observed between the tip and the center of the cucumber, suggesting that there is no bias in the distribution of antioxidants between the center and the tip of the cucumber. Next, fluorescence images were taken for the shoulder and tip sections of carrots after GQDs and Fe^3+^ were dropped onto them. Comparing the shoulder section and the tip section, stronger fluorescence was observed in the tip section than in the shoulder section, suggesting that antioxidants are distributed at higher concentrations in the tip section of carrots. However, to further prove these findings, several cucumber and carrot samples were imaged. As shown in Figure 8, the different samples behaved similarly, which confirms the reliability of the method for imaging experiments. In addition, similar results were obtained in the chemiluminescence imaging method of antioxidants developed in our laboratory in the past [25].

Afterward, evaluation of antioxidant activity, as mg ascorbic acid equivalent/100 g sample, for crushed cucumber (central and tip) and carrot (shoulder and tip) extracts was conducted using the paper-based assay, and the obtained results were compared with those obtained by the imaging of the intact sections. Since solvent extraction of food components using water as a solvent is easy, aqueous extracts of foods are widely used for the evaluation of antioxidant capacity. Therefore, the paper-based fluorescence imaging method was applied to evaluate the antioxidant capacity in cucumber and carrot aqueous extracts.

The antioxidant capacity of cucumber and carrot extracts evaluated by fluorescence imaging was converted into l-ascorbic acid equivalents (mgASC/100 g sample) and these are summarized in Table 8. Additionally, these extracts were analyzed by another reported method [25], and the obtained results were not significantly different, as proved by the value of the paired student’s *t*-test (*p* = 0.2848) and the obtained high correlation coefficient (r = 0.9984).

In the case of cucumber, there is no significant difference in antioxidant capacity between the tip and the center of the cucumber, while in the case of carrot, the antioxidant capacity is higher in the tip of the carrot than in the shoulder. This result is consistent with the results obtained from the analysis of fluorescent images of carrots shown in Figure 8. Thus, this fluorescence imaging method is useful for quantifying the antioxidant capacity of food extracts and is considered to be useful for visualization and analysis of the distribution of antioxidants in food samples.

## 4. Conclusions

A novel fluorometric approach for antioxidant assay utilizing the fluorescence quenching of GQDs by Fe^3+^ and the fluorescence recovery effect of antioxidants was developed. The method was applied successfully for the accurate and precise determination of several antioxidants. Fluorescence imaging of antioxidants was performed using Fe^3+^-GQDs and ChemiDoc imaging systems. The antioxidant capacity of the vitamin C supplement beverage was successfully calculated using the imaging technique, and the values found matched the manufacturer’s claim on the label. Finally, fluorescence imaging of the antioxidant distribution profile on slices of cucumber and carrot tips, center, and shoulder was performed, and the results were consistent with the antioxidant capacity of these parts of aqueous extracts.

## Figures and Tables

**Table 1:** Calibration curves and LOD of the tested l-ascorbic acid at different concentrations of Fe^3+^.

Concentration of Fe^3+^ (mM)	l-Ascorbic Acid Range (µM)	Calibration Equation for l-Ascorbic Acid *	Coefficient of Determination (r^2^)	LOD of l-Ascorbic Acid (µM) **
0.06	2.0–5.0	Y = 18.5X + 466	0.986	0.222
0.12	2.0–15.0	Y = 11.9X + 449	0.968	0.512
0.3	5.0–50.0	Y = 6.18X + 395	0.998	0.805
0.6	5.0–100	Y = 4.04X + 383	0.990	1.60

* Y = fluorescence intensity; X = concentration of l-ascorbic acid, µM; ** limit of detection (3SD/slope).

**Table 2:** Calibration curves and LOD of the tested antioxidants.

Antioxidant	Linear Range (µM)	Calibration Equation *	r^2^	LOD ** (µM)
l-Ascorbic acid	5.0–50.0	Y = 6.18X + 395	0.998	0.805
Gallic acid	2.0–20.0	Y = 10.8X + 429	0.992	0.460
Pyrogallol	5.0–20.0	Y= 8.24X + 414	0.995	0.603
DTT	5.0–50.0	Y = 3.54X + 448	0.999	1.40
(+)-Catechin	10.0–50.0	Y= 2.74X + 474	0.996	1.81
Caffeic acid	20.0–100	Y = 0.604X + 485	0.998	8.23
Trolox	5.0–50.0	Y = 5.92X + 566	0.991	2.49

* Y = fluorescence intensity; X = concentration of antioxidants, µM; ** limit of detection (3SD/slope).

**Table 3:** Accuracy and precision of the proposed spectrofluorometric method.

Antioxidant	Concentration (µM)	Accuracy	Precision (%RSD)	
			Within Day (*n* = 5)	Between Days (*n* = 5)
l-Ascorbic acid	5.0	90.6	5.4	3.5
	20.0	88.47	2.6	2.9
	50.0	86.08	2.3	1.8
Gallic acid	5.0	103.26	4.9	4.2
	10.0	100.33	2.4	2.9
	20.0	98.63	6.7	2.0
Pyrogallol	5.0	108.97	4.8	2.0
	10.0	103.5	3.9	2.0
	20.0	108.55	3.5	1.7
DTT	5.0	115.8	8.1	3.0
	20.0	113.34	7.7	1.9
	50.0	110.76	4.7	2.3
(+)-Catechin	10.0	111.5	5.3	2.9
	20.0	113.8	8.3	3.6
	50.0	113.75	6.6	2.0
Caffeic acid	30.0	105.87	3.3	3.1
	50.0	113.8	5.5	1.4
	100.0	111.8	7.3	2.5
Trolox	5.0	93.37	6.5	3.6
	20.0	102.94	5.4	2.5
	50.0	92.6	4.1	3.9

**Table 4:** Determination of total antioxidant content in lemon and apple juices.

Sample	Parameter	Value
Lemon juice	Antioxidant content (mM ASC; mean found ± SD)	
	Proposed method	12.59 ± 0.52
	Comparison method [44]	12.65 ± 0.29
	N	3
	*t*-test (2.77)	0.15
	F-test (19.00)	3.21
Apple juice	Antioxidant content (mM ASC; mean found ± SD)	
	Proposed method	0.544 ± 0.041
	Comparison method [44]	0.529 ± 0.035
	N	3
	*t*-test (2.77)	0.49
	F-test (19.00)	1.37

**Table 5:** Comparison of sensitivity between the proposed method and reported methods.

Antioxidant	LOD (µM)		
	Proposed Method	FRAP Method [45]	Chemiluminescence Sensing [25]
l-Ascorbic acid	0.805	14.2	0.18
Gallic acid	0.460	77.6	1.19
Pyrogallol	0.603		0.28
DTT	1.40		0.61
(+)-Catechin	1.81	0.517	
Trolox	2.49	12.3	

**Table 6:** Accuracy and precision of the proposed paper-based assay method.

l-Ascorbic Acid Concentration (µM)	Accuracy	Precision (%RSD)	
		Within Day (*n* = 5)	Between Days (*n* = 5)
100.0	115.62	8.2	7.9
1000.0	93.63	3.0	3.4
2000.0	101.62	7.9	2.1

**Table 7:** Concentration of l-ascorbic acid in a vitamin C drink.

Parameter	Reported (μM)	Found (μM)	Found (%) *
	500	479.6	95.9
	1000	921.0	92.1
	1500	1444	96.3
Mean ± SE (%)	94.8 ± 5.83		
Nominal (mM)	38.45 ± 2.36		

* Each result is the average of five separate determinations.

**Table 8:** The proposed method was applied to the determination of the antioxidant capacities of food samples.

Sample	Antioxidant Capacity (mg ASC/100 g Sample) Mean ± SD Using FL Method	Antioxidant Capacity (mg ASC/100 g Sample) Using the Reference Method [25]
Cucumber		
Central part	252.5 ± 13.1	254.6 ± 10.4
Tip	256.9 ± 26.9	260.6 ± 27.5
Carrot		
Near shoulder	227.2 ± 9.2	225.1 ± 8.1
Tip	276.2 ± 17.8	279.3 ± 21.2
Paired *t*-test *p*-value	0.2848	
r	0.9984	

## Data Availability

Most of the data are shown in the original manuscript, and if further data is needed, it will be available upon reasonable request.
